# Upper hemisternotomy vs. full sternotomy for hemiarch and proximal aortic replacement

**DOI:** 10.3389/fcvm.2025.1646209

**Published:** 2026-01-30

**Authors:** Jeffrey A. Zucker, Vishal N. Shah, Joshua R. Chen, Christopher Pritting, Colin King, Jacqueline McGee, Megary McCoy, Konstadinos Plestis

**Affiliations:** Department of Cardiothoracic Surgery, Thomas Jefferson University Hospital, Philadelphia, PA, United States

**Keywords:** upper hemisternotomy, hemiarch, proximal aortic replacement, antegrade cerebral perfusion, retrograde, myocardial protection

## Abstract

**Introduction:**

Hemiarch replacement with proximal aortic replacement is seldom performed via upper hemisternotomy (UHS). We report our surgical technique and postoperative outcomes in 11 patients who underwent UHS for hemiarch and proximal aortic replacement, compared with 15 patients who underwent the procedure via full sternotomy (FS).

**Methods:**

A UHS was performed at the right third or fourth intercostal space. Cardiopulmonary bypass (CPB) was established via the distal ascending aorta or right axillary artery and right common femoral vein. After aortic cross-clamping (ACC), the heart was arrested with single-dose antegrade crystalloid cardioplegia. After proximal aortic replacement, hypothermic circulatory arrest (HCA) between 20 and 24 °C was initiated with bilateral antegrade or retrograde cerebral perfusion, and hemiarch replacement performed.

**Results:**

Between February and December 2010, 15 patients (median age 67 yr) underwent hemiarch repair with proximal aortic replacement using FS. From April 2015 to February 2019, 11 patients (median age 74 yr) underwent the same procedure via UHS. Median CPB, ACC, and HCA times were 192 min vs. 185 min (*P* = 0.72), 105 min vs. 157 min **(*P*** **=** **0.03)**, and 5 min vs. 15 min (*P* = 0.95) for UHS and FS, respectively. There were no in-hospital deaths. Survival at 1 and 5 yr was 100% and 72.7% in the UHS group, and 100% and 80% in the FS group (*P* = 0.13, *P* = 1.0).

**Conclusions:**

Low morbidity and mortality demonstrate that UHS for combined hemiarch and proximal aortic replacement is safe and feasible. Larger studies are needed to confirm these findings.

## Introduction

Over the past several decades, less invasive approaches to aortic valve replacement (AVR) including upper hemisternotomy (UHS) have become popular (1–4). Recently, UHS has demonstrated safety and feasibility in patients undergoing complex proximal aortic surgery (5). We have previously reported similar or, in some cases, superior outcomes with UHS compared to full sternotomy (FS) for composite valve graft aortic root replacement (ARR; i.e., Bentall procedure), valve-sparing ARR (i.e., David procedure), and aortic valve replacement (AVR) combined with supracoronary ascending aorta replacement (scAAR) (6–9). Nevertheless, UHS is seldom used for combined hemiarch and proximal aortic replacement because of the added technical complexity, intraoperative decision-making challenges, and concerns regarding adequate surgical exposure. Consequently, we present our UHS approach, highlight key technical aspects, and report outcomes in 11 patients undergoing elective hemiarch and proximal aortic replacement via UHS compared with 15 patients undergoing FS.

## Methods

### Selection criteria

Between February and December 2010, 15 patients underwent elective hemiarch and proximal aortic replacement via FS for aneurysmal disease. From April 2015 through February 2019, 11 consecutive patients underwent the same procedure for aneurysmal disease using a J-type UHS. All procedures were performed by K.P., the senior author (Table 1). Patients who required urgent or emergent procedures or who had a concomitant cardiac procedure other than hemiarch replacement [i.e., coronary artery bypass grafting (CABG) or mitral valve surgery] or who required total arch replacement (TAR) using hypothermic circulatory arrest (HCA), or repair of acute type A aortic dissection (ATAAD), reoperation, or root abscess were excluded from this study. To mitigate selection bias between UHS and FS patients, exclusion criteria were established based on procedures not performed on UHS patients by the senior author. Patients were identified from a prospectively maintained aortic registry, and their data were studied retrospectively. Mortality was assessed using an internal database repository, queries to the Social Security Death Index, and online obituary searches. The Institutional Review Board approved the study (iRISID-2023-2730) on January 8, 2024. The need for informed consent was waived due to the study's retrospective nature.

**Table 1:** Preoperative demographics.

Preoperative characteristics	**UHS *n*** **=** **11**	** FS *n*** **=** **15**	***P*-value**	**Total *n*** **=** **26**
Male gender	4 (36.4)	7 (46.7)	0.90	11 (42.3)
Age (yr)	74 (60–78.5)	67 (52–71.5)	0.07	68 (53–75.5)
Hypertension	8 (72.7)	11 (73.3)	1.00	19 (73.1)
Hyperlipidemia	8 (72.7)	7 (46.5)	0.35	15 (57.7)
Diabetes	2 (18.2)	1 (6.7)	0.77	3 (11.5)
COPD	2 (18.2)	2 (13.3)	1.00	4 (15.4)
Prior stroke	0 (0.0)	0 (0.0)	—	0 (0.0)
Prior TIA	0 (0.0)	0 (0.0)	—	0 (0.0)
Renal insufficiency	0 (0.0)	0 (0.0)	—	0 (0.0)
Congestive heart failure	2 (18.2)	0 (0.0)	0.43	2 (0.8)
Ejection fraction	65 (54–67.5)	60 (59.5–63.5)	0.39	60.5 (56–65)

Continuous data presented as median with interquartile range (IQR). Categorical data presented as frequency (%). COPD, chronic obstructive pulmonary disease; FS, full sternotomy; TIA, transient ischemic attack; UHS, upper hemisternotomy.

### Data analysis

The primary outcomes were in-hospital mortality and 1 and 5 yr survival rates. Secondary outcomes included stroke, transient ischemic attack (TIA), renal insufficiency, prolonged ventilatory support (PVS), reoperation for bleeding, cardiopulmonary bypass (CPB), aortic cross-clamp (ACC) and HCA times, and hospital length of stay (LOS). Stroke was defined as loss of neurological function with residual symptoms at least 72 h after onset, while TIA was defined as loss of neurological function that was abrupt onset but with full recovery of function within 24 h. Renal insufficiency was defined as doubling the serum creatinine or a new requirement for hemodialysis and PVS was defined as the need for >48 h of mechanical ventilation postoperatively. Other complications were defined according to the Society of Thoracic Surgeons Adult Cardiac Surgery Database specifications.

### Surgical technique

#### Preoperative assessment

Transthoracic and intraoperative transesophageal echocardiography (TEE) was utilized to inspect valve morphology and dysfunction, severity of aortic insufficiency (AI), presence of cusp calcification or prolapse, and ventricular size and function. Computed tomography angiography (CTA) with 3D reconstructions was used to evaluate the morphology and dimensions of the aortic root, ascending aorta, and proximal aortic arch. Specific emphasis was placed on CTA to assess the locations of the aortic annulus and the right internal mammary artery (RIMA), as the UHS terminates one intercostal space (ICS) above the plane of the aortic annulus—typically corresponding to the third or fourth ICS. Typically, scAAR and/or ARR procedures were performed when the ascending aortic or aortic root diameter reached ≥5 cm, or ≥4.5 cm in patients with a bicuspid aortic valve. When ARR was not indicated, AVR was performed in patients with moderate or severe AI. When the aortic arch diameter was ≥4 cm at the level of the innominate artery takeoff or beyond, and the dilation was confined to the lesser curvature of the aortic arch, hemiarch or extended hemiarch replacement was indicated. If the aneurysm was limited to the proximal side of the innominate artery origin, an open distal anastomosis between the distal end of the graft and the proximal aortic arch on the lesser curvature at the level of the innominate artery was performed (i.e., hemiarch replacement). If the aneurysm extended distal to the origin of the innominate artery but remained confined to the lesser curvature of the aortic arch, the diseased segment of the arch was excised along the lesser curvature without manipulation of the head vessels. The distal anastomosis was sewn opposite the origin of the left carotid or left subclavian artery (i.e., extended hemiarch replacement). Pulmonary function testing and coronary angiography were also performed in all patients.

#### Incision and cannulation

**Figure 1:**
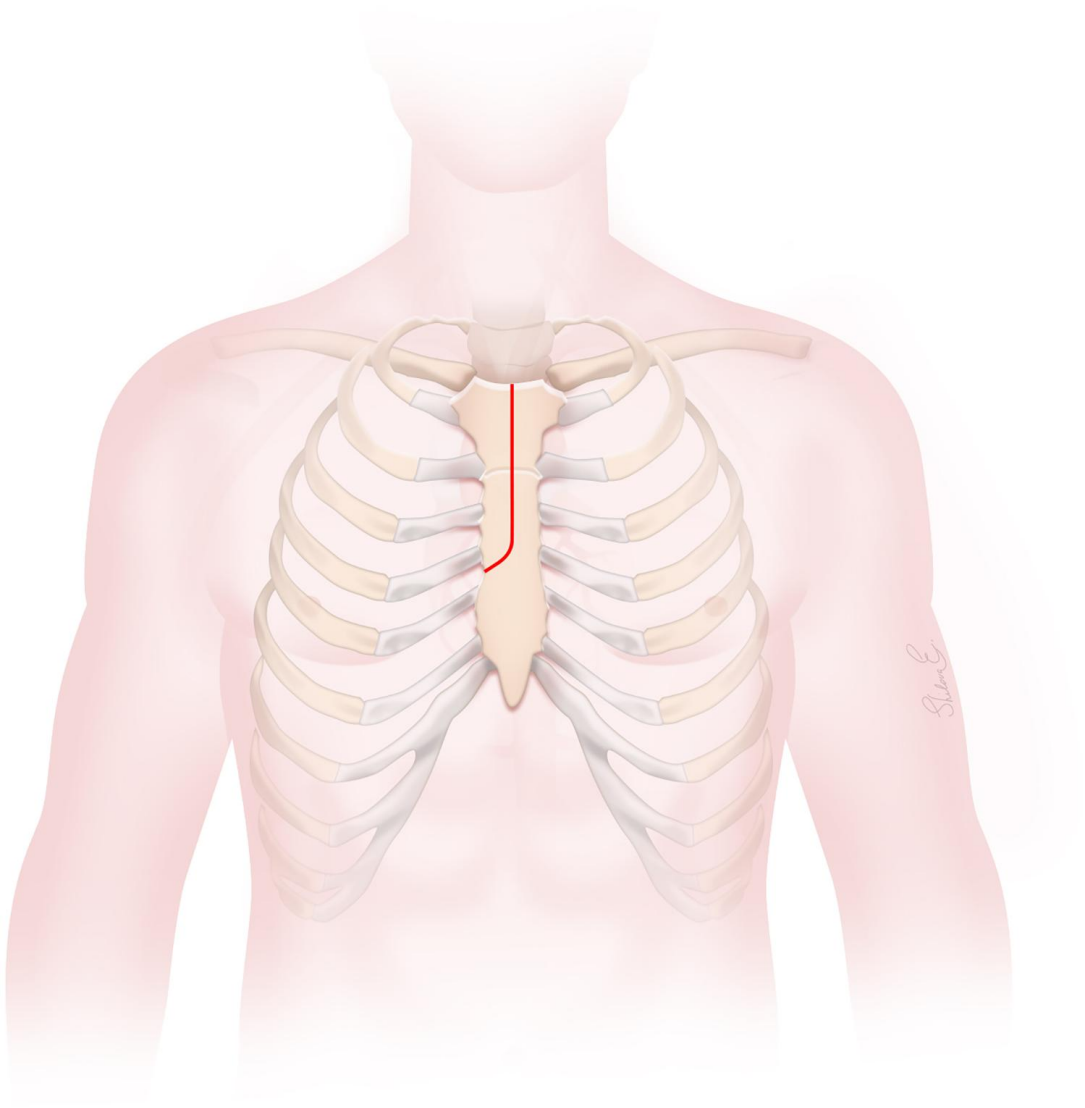
The J-type upper hemisternotomy incision.

**Figure 2:**
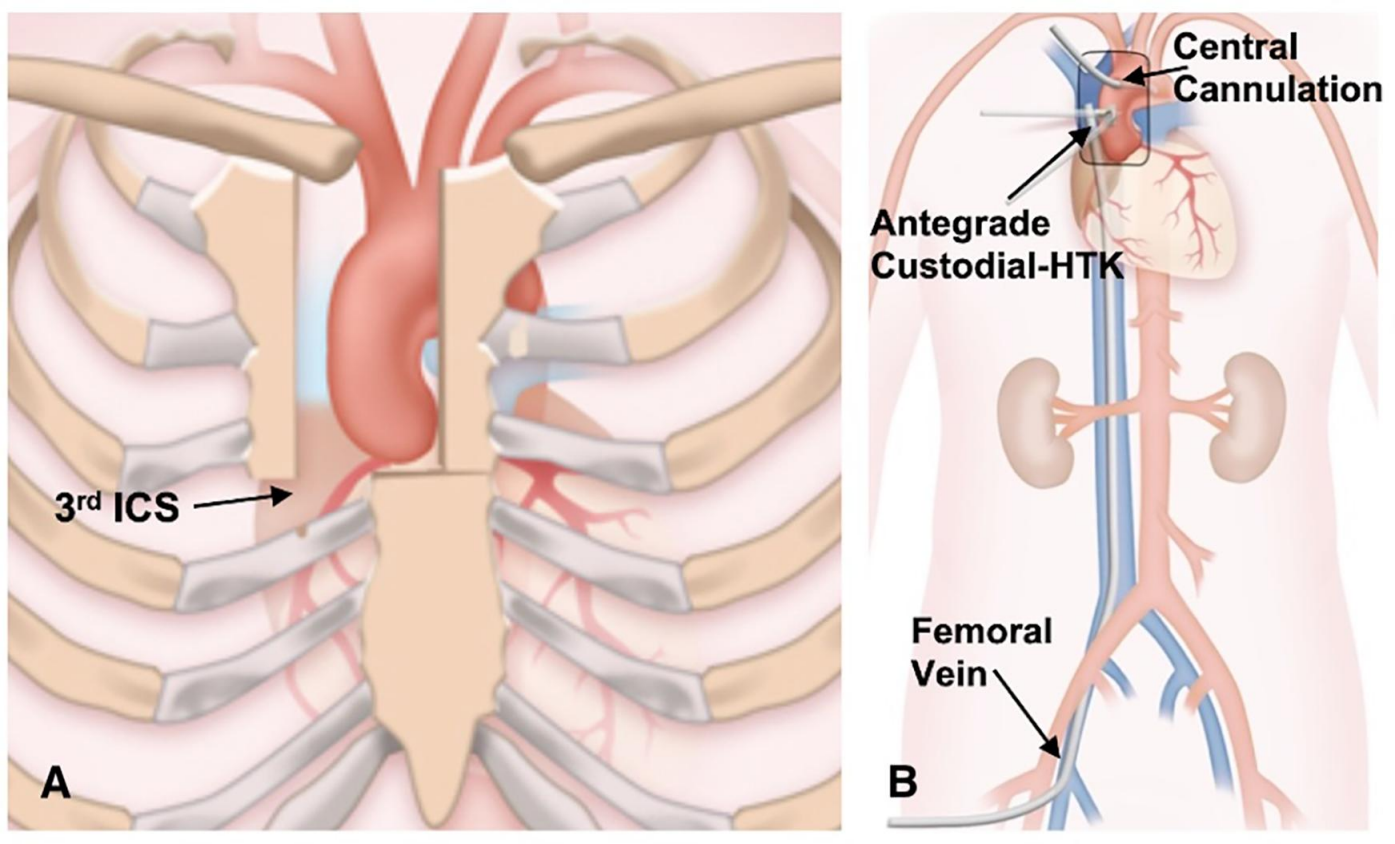
**(A)** the J-type upper hemisternotomy exiting the right third intercostal space. **(B)** Cannulation strategy for upper hemisternotomy.

A J-type UHS was performed, extending from 2 cm below the sternal notch to the midpoint of the third or fourth ICS and exiting at the right lateral third or fourth ICS, carefully avoiding the RIMA (Figures 1, 2A). Adjunctive techniques to enhance exposure during UHS included the strategic placement of pericardial traction sutures to anteriorize and medialize the ascending aorta, as well as cephalad traction sutures around the ACC and through the pericardium. In patients with a deep mediastinum, pericardial sutures were placed deeply, the sternal retractor was removed, upward traction was applied to the pericardial sutures, and the retractor was subsequently reinserted. When using retrograde cerebral perfusion (RCP), the proximal aortic arch along the lesser curvature was directly cannulated using the Seldinger technique under TEE guidance (Figure 2B). When using bilateral antegrade cerebral perfusion (bACP), the right axillary artery was exposed via an infraclavicular incision. Subsequently, a 10 mm Dacron graft was anastomosed end-to-side to the axillary artery. The right femoral vein was directly cannulated using a small incision, and a 25 Fr long femoral venous cannula (Bio-Medicus; Medtronic, Minneapolis, MN, USA) was advanced into the superior vena cava using the Seldinger technique under TEE guidance. The femoral venous cannula was “Y”-ed to a smaller right atrial cannula if additional drainage was needed. During FS, the right atrium was cannulated directly. CPB flows were maintained at 2.4 L/min/m^2^ at normothermia and 1.8 L/min/m^2^ during cooling, with goal mean arterial pressures between 60 and 70 mm Hg. Adequate cerebral cooling was confirmed by jugular venous oxygen saturation higher than 95% and a bladder temperature of 20–24 °C. The head was packed with ice. The cooling process lasted at least 30 min with confirmation of electrocerebral silence on the electroencephalogram. After aortic cross-clamping (ACC), 2 L of Custodiol histidine–tryptophan–ketoglutarate (HTK; Essential Pharmaceuticals LLC, Durham, NC, USA) was administered into the aortic root or directly into the coronary ostia. Custodiol-HTK cardioplegia provides myocardial protection for up to 2–3 h with a single dose. When there was severe AI, an initial 1 L dose of antegrade cardioplegia was infused, and the remaining solution was then instilled directly into the coronary ostia after aortotomy. During FS, conventional cold blood potassium-based cardioplegia was administered, with repeated doses given every 15–20 min. During UHS, a direct main pulmonary artery vent was typically inserted, whereas during FS, a right superior pulmonary vein vent was generally employed. Carbon dioxide insufflation was used in all patients.

#### Procedure

**Figure 3:**
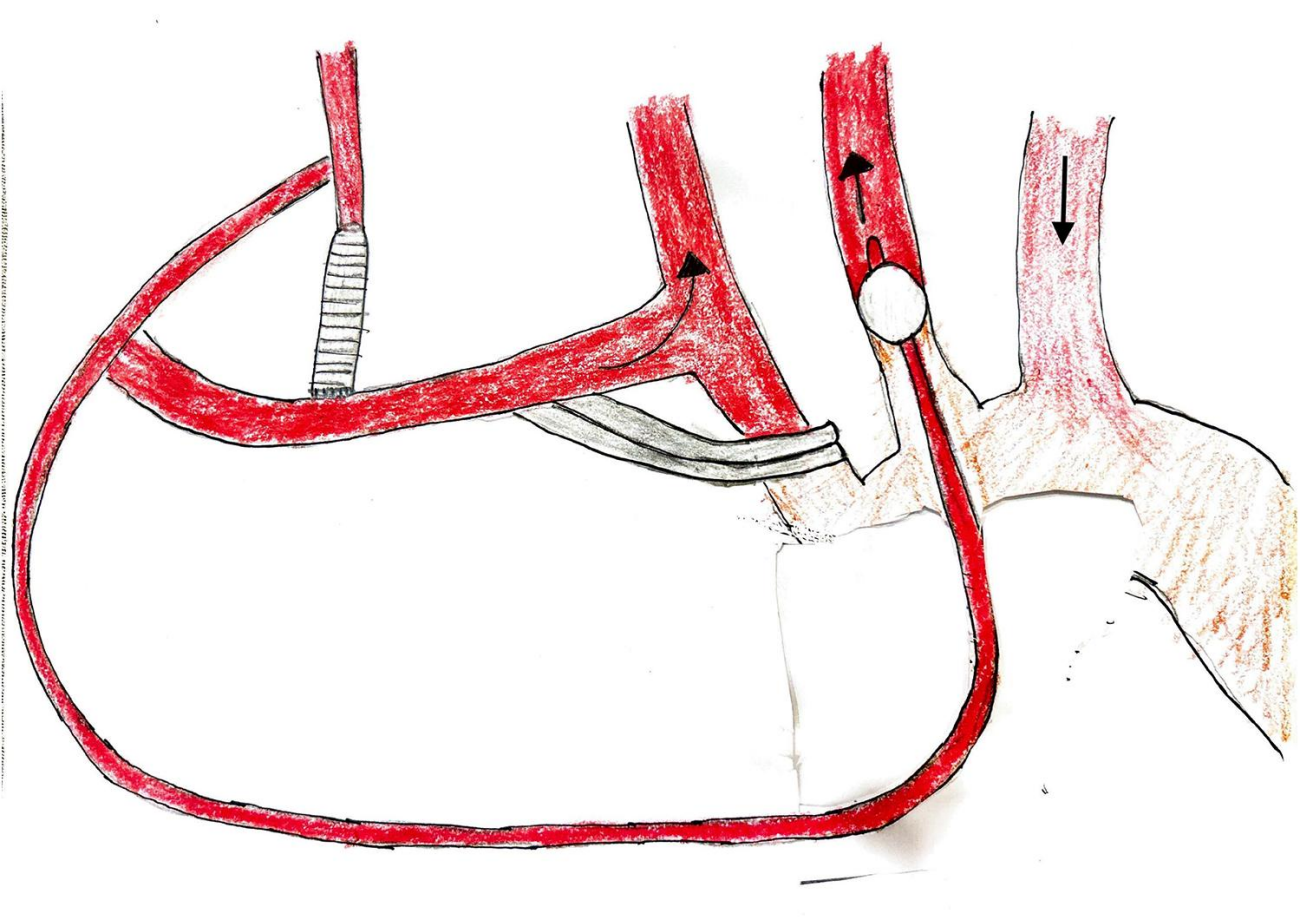
Bilateral antegrade cerebral perfusion setup.

Our technique for AVR, ARR, and combined AVR and scAAR procedures has been described previously (6–8). During cooling, the proximal aortic replacement was done. In UHS cases, the COR-KNOT® automated suture fastener (LSI SOLUTIONS, Victor, NY, USA) was used to secure the composite valve graft in Bentall procedures and the Valsalva graft in David procedures. Hand-tied knots were used in non-ARR UHS cases and in all FS cases. Three minutes before HCA, intravenous bolus doses of 1 g methylprednisolone, 100 mg phenytoin, and 50–100 mg propofol were administered. After reaching adequate hypothermia (20–24 °C), the patient was placed in Trendelenburg, the vent was clamped, and circulatory arrest was initiated. The bACP was established by clamping the innominate artery 1 cm above its origin and placing a 9 Fr Pruitt perfusion balloon-tip catheter (LeMaitre Vascular, Inc, Burlington, MA) “Y”-ed to the arterial line into the left common carotid artery (Figure 3). The RCP was established by inserting a coronary sinus catheter into the superior vena cava and snaring the vessel. The cerebral perfusion strategy was determined by the location of the distal anastomosis, particularly during UHS. We use bACP for extended hemiarch replacements, and RCP for hemiarch replacements during UHS. The proximal aortic arch was transected in a beveled fashion, and the main graft was sewn to the undersurface of the aortic arch. The location of the distal anastomosis was based on the extent of the aneurysm at the innominate artery takeoff or beyond confined to the lesser curvature of the aortic arch. The distal anastomosis was constructed in all patients using a continuous 4–0 polypropylene suture with a strip of Teflon felt incorporated. The entire anastomosis was then reinforced in all patients with Teflon felt pledgets placed externally to provide a secondary hemostatic layer. After the distal anastomosis was completed, the innominate artery clamp was removed, the graft was de-aired, and the main graft was clamped. When using RCP, the arterial cannula was placed into the side branch of the main graft. The graft was then de-aired, and the main graft was clamped. Subsequently, CPB and rewarming were initiated. The remainder of the proximal aortic replacement was completed if not already done. The patient was weaned from CPB. The UHS was closed with a combination of steel wires and rigid plates.

### Statistical analysis

After descriptive analysis of baseline patient characteristics, comparative analyses were performed between the UHS and FS groups, followed by survival analysis. Categorical variables were summarized as proportions and compared using the *χ*^2^ test. Continuous variables were reported as medians with interquartile ranges (IQRs) and compared using the Kruskal–Wallis test. Survival estimates were calculated using the Kaplan–Meier method, and subgroup comparisons were assessed with the log-rank test. A *P*-value < 0.05 was considered statistically significant. All analyses and visualizations were conducted using R software, version 4.3.3 (R Foundation for Statistical Computing, Vienna, Austria).

## Results

Baseline demographics of the 11 UHS and 15 FS patients are summarized in Table 1. The median age was 74 yr (IQR, 60–78.5 yr) in the UHS group and 67 yr (IQR, 52–71.5 yr) in the FS group (*P* = 0.07). Both groups demonstrated a female preponderance (UHS: 7/11, 63.6%; FS: 8/15, 53.3%; *P* = 0.90). Hypertension was the most common comorbidity (UHS: 8/11, 72.7%; FS: 11/15, 73.3%; *P* = 1.00). In the UHS group, 5 patients (45.5%) had moderate or severe AI, 2 patients (18.2%) had mild AI, and 4 patients (36.4%) had no AI, whereas in the FS group, 10 patients (66.7%) had moderate or severe AI, 3 patients (20%) had mild AI, and 2 patients (13.3%) had no AI (*P* = 0.58) (Table 2). In the UHS cohort, 7 patients (63.6%) received bACP and 4 patients (36.4%) received RCP. In comparison, in the FS cohort, 9 patients (60%) received no cerebral perfusion, 5 patients (33.3%) received bACP, and 1 patient (6.7%) received RCP (*P* < 0.01). Table 2 summarizes the types of procedures performed in UHS and FS patients, with no statistically significant differences between the groups (*P* = 0.05). The median diameters of the aortic root, ascending aorta, and aortic arch for the UHS and FS cohorts are shown in Table 2.

**Table 2:** Intraoperative outcomes.

Intraoperative outcomes	**UHS *n*** **=** **11**	** FS *n*** **=** **15**	***P*-value**	**Total *n*** **=** **26**
Aortic measurements				
Aortic root diameter (cm)	3.8 (3.5–4.2)	4 (3.9–4.5)	0.10	3.9 (3.7–4.4)
Ascending aortic diameter (cm)	5.5 (5–5.8)	5 (4.9–5.2)	0.07	5.1 (5–5.7)
Aortic arch diameter (cm)	3.9 (3.8–4.6)	3.5 (3.3–4)	0.12	3.9 (3.4–4.3)
Degree of preoperative aortic insufficiency				
None	4 (36.4)	2 (13.3)	0.58	6 (23.1)
Mild	2 (18.2)	3 (20)		5 (19.2)
Moderate	3 (27.3)	6 (40)		9 (34.6)
Severe	2 (18.2)	4 (26.7)		6 (23.1)
Arterial inflow				
Distal ascending aorta	4 (36.4)	4 (26.7)	0.76	8 (30.8)
Right axillary artery	7 (63.6)	11 (73.3)		18 (69.2)
Venous drainage				
Right common femoral vein	11 (100)	0 (0.0)	**<0.01**	11 (42.3)
Right atrium	0 (0.0)	15 (100)		15 (57.7)
Procedure type				
Hemiarch replacement + scAAR	1 (9.1)	1 (6.7)	0.05	2 (7.7)
Hemiarch replacement + scAAR + aortic valve repair	0 (0.0)	1 (6.7)		1 (3.9)
Hemiarch replacement + scAAR + AVR	0 (0.0)	1 (6.7)		1 (3.9)
Hemiarch replacement + scAAR + David	2 (18.2)	1 (6.7)	—	3 (11.5)
Hemiarch replacement + scAAR + Bentall	1 (9.1)	6 (40.0)		7 (26.9)
Extended hemiarch replacement + scAAR	6 (54.5)	0 (0.0)	—	6 (23.1)
Extended hemiarch replacement + scAAR + aortic valve repair	1 (9.1)	1 (6.7)	—	2 (7.7)
Extended hemiarch replacement + scAAR + AVR	0 (0.0)	2 (13.3)	—	2 (7.7)
Extended hemiarch replacement + scAAR + Bentall	0 (0.0)	2 (13.3)	—	2 (7.7)
Cerebral perfusion type				
None	0 (0.0)	9 (60.0)	**<0.01**	9 (34.6)
Bilateral antegrade	7 (63.6)	5 (33.3)		12 (46.2)
Retrograde	4 (36.4)	1 (6.7)		5 (19.2)
Procedural times				
CPB (min)	192 (169–211)	185 (162–210)	0.72	188.5 (168–211)
ACC (min)	105 (93–128)	157 (135–174)	**0**.**03**	142 (106–167)
HCA (min)	5 (2–29)	15 (11–18)	0.95	14.5 (5–20)
Blood product transfusions				
PRBC (U)	3 (1–4.5)	2 (0.5–4.5)	0.67	3 (0.2–4.8)
FFP (U)	4 (1.5–6)	3 (2–4)	0.57	3.5 (2–4)
Platelets (U)	3 (1.5–4)	0 (0–10)	0.39	1.5 (0–4)
Cryoprecipitate (U)	2 (1–4)	2 (1.5–3)	0.65	2 (1.2–3.8)
Cardioplegia type				
Custodiol-HTK cardioplegia	11 (100)	0 (0.0)	**<0.01**	11 (42.3)
Blood cardioplegia	0 (0.0)	15 (100)		15 (57.7)
Knot-tying type				
Hand-tied	8 (72.7)	15 (100)	0.13	23 (88.5)
COR-KNOT®	3 (27.3)	0 (0.0)		3 (11.5)

Continuous data presented as median with interquartile range (IQR). Categorical data presented as frequency (%). ACC, aortic cross clamp; AVR, aortic valve replacement; CPB, cardiopulmonary bypass; FFP, fresh frozen plasma; FS, full sternotomy; HCA, hypothermic circulatory arrest; HTK, histidine–tryptophan–ketoglutarate; PRBC, packed red blood cell; scAAR, supracoronary ascending aorta replacement; UHS, upper hemisternotomy.

Bolded text indicates statistically significant findings.

Intraoperative details are summarized in Table 2. For arterial inflow, the distal ascending aorta was cannulated in 4 UHS patients (36.4%) and 4 FS patients (26.7%), whereas the right axillary artery was used in 7 UHS patients (63.6%) and 11 FS patients (73.3%) (*P* = 0.76). Venous drainage was achieved via the right common femoral vein in all UHS patients (11/11, 100%), and via direct right atrial cannulation in all FS patients (15/15, 100%) (*P* < 0.01). The median CPB, ACC, and HCA times were 192 min (IQR, 169–211) vs. 185 min (IQR, 162–210; *P* = 0.72), 105 min (IQR, 93–128) vs. 157 min (IQR, 135–174; *P* = 0.03), and 5 min (IQR, 2–29) vs. 15 min (IQR, 11–18; *P* = 0.95) in the UHS and FS cohorts, respectively. Table 2 presents the intraoperative blood product transfusion requirements for UHS and FS procedures. No cases required conversion from UHS to FS. From 2013 onward, the use of COR-KNOT® and Custodiol-HTK cardioplegia became routine in all cases, following a change in surgeon preference. Cold blood potassium-based cardioplegia was used in all FS patients (15/15, 100%), whereas Custodiol-HTK cardioplegia was used in all UHS patients (11/11, 100%; *P* < 0.01). Hand-tied knots were employed in all FS cases (15/15, 100%), while COR-KNOT® was used in 3 UHS cases (33.3%) to secure the composite valve or Valsalva graft during ARR (*P* = 0.13; Table 2).

**Figure 4:**
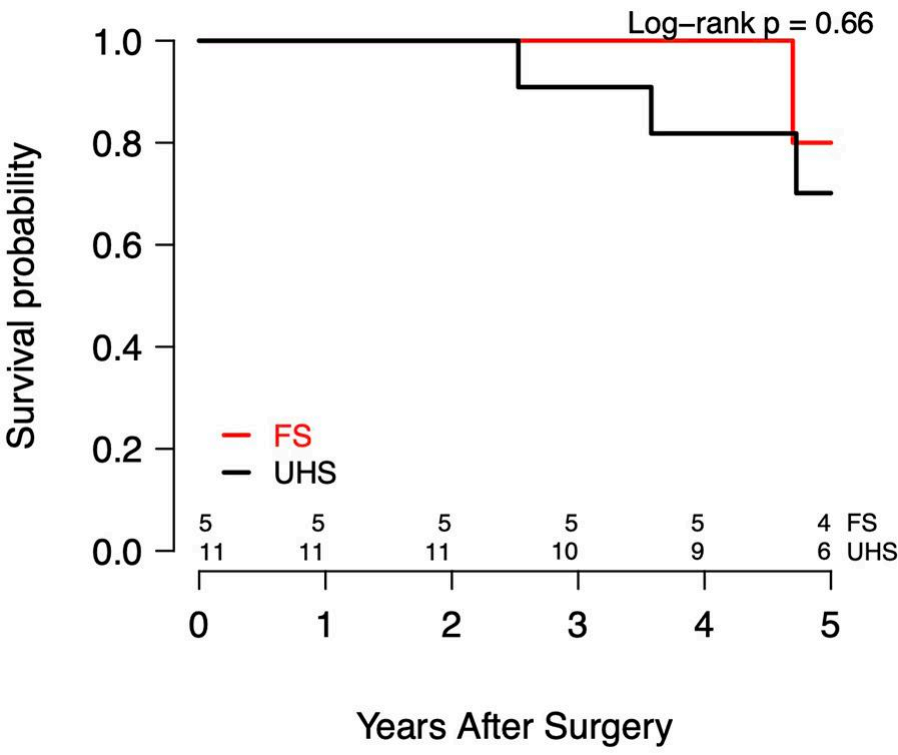
Kaplan–meier survival curves.

There were no in-hospital deaths in either cohort. As summarized in Table 3, which reports on the postoperative outcomes, there were no postoperative strokes, TIAs, renal insufficiency, or reoperations for bleeding in either group. The hospital LOS was 7 d (IQR, 6–11 d) for UHS patients and 8 d (IQR, 7–13 d) for FS patients (*P* = 0.51). Among 11 patients with follow-up in the UHS cohort, overall survival was 100% at 1 yr and 72.7% at 5 yr (*P* = 0.13). In the FS cohort, which included 5 patients with follow-up, overall survival was 100% at 1 yr and 80.0% at 5 yr (*P* = 1.00) (Table 4). The Kaplan–Meier survival curves for both cohorts are shown in Figure 4. There were no reinterventions or distal aortic events after UHS.

**Table 3:** Postoperative outcomes.

Postoperative outcomes	**UHS *n*** **=** **11**	**FS *n*** **=** **15**	***P*-value**	**Total *n*** **=** **26**
In-hospital mortality	0 (0.0)	0 (0.0)	—	0 (0.0)
ICU LOS (d)	4 (3–5)	4 (2.5–7)	0.98	4 (3–5)
Hospital LOS (d)	7 (6–11)	8 (7–13)	0.51	8 (6.2–11.8)
Stroke	0 (0.0)	0 (0.0)	—	0 (0.0)
TIA	0 (0.0)	0 (0.0)	—	0 (0.0)
PVS	3 (27.3)	4 (26.7)	1.00	7 (26.9)
Renal insufficiency	0 (0.0)	0 (0.0)	—	0 (0.0)
Re-intubation	0 (0.0)	0 (0.0)	—	0 (0.0)
Reoperation for bleeding	0 (0.0)	0 (0.0)	—	0 (0.0)
Atrial fibrillation	5 (45.5)	3 (20)	0.34	8 (30.8)

Continuous data is presented as median with interquartile range (IQR). Categorical data is presented as frequency (%). FS, full sternotomy; ICU, intensive care unit; LOS, length of stay; PVS, prolonged ventilatory support; TIA, transient ischemic attack; UHS, upper hemisternotomy.

**Table 4:** Survival outcomes.

Survival outcomes	**UHS *n*** **=** **11**	** FS *n*** **=** **5**	***P*-value**
Follow-up (yr)	5.0 (4.5–6.1)	15.1 (15.0–15.2)	**0**.**02**
1 yr survival	11 (100)	5 (100)	0.13
5 yr survival	8 (72.7)	4 (80)	1.00

Continuous data is presented as median with interquartile range (IQR). Categorical data is presented as frequency (%). FS, full sternotomy; UHS, upper hemisternotomy.

Bolded text indicates statistically significant findings.

## Discussion

The UHS approach for minimally invasive aortic surgery is a safe and reproducible alternative to the FS approach. A recent large meta-analysis comparing minimally invasive aortic root surgery with FS reported lower rates of reoperation for bleeding, renal impairment, ICU and hospital LOS, and CPB time with the minimally invasive approach, without a significant difference in mortality (10). These findings were confirmed by Kim et al., who reported low early mortality, low FS conversion rates, and a low incidence of major postoperative complications following mini-access open arch repair (11). Additionally, Helms et al., like us, found that minimally invasive proximal aortic surgery is safe, with perioperative and long-term complication rates comparable to conventional FS (12).

In our small cohort, there were no in-hospital deaths, consistent with larger studies reporting low mortality rates of approximately 3%–4%, particularly following FS hemiarch and proximal aortic replacement (13, 14). Malaisrie et al. reported that, among 133 propensity score–matched patients undergoing FS hemiarch and proximal aortic replacement, adding hemiarch replacement to proximal aortic surgery did not increase mortality risk (14). Our 5 yr UHS survival rate was lower than that reported for the FS cohort by Malaisrie et al. (72.7% vs. 88%), likely related to the older age of our cohort (74 vs. 56.2 yr) (14). No reoperations occurred during UHS follow-up, owing to complete excision of all aneurysmal tissue.

Our experience demonstrates low morbidity, consistent with other UHS series. In 44 patients undergoing hemiarch replacement via UHS, with ARR in 19 and AVR in 21, Kim et al. reported 1 operative death (2.3%), 2 reoperations for bleeding (4.5%), and 1 conversion to FS (2.3%) (11). Similarly, Goebel et al. reported their initial experience with UHS aortic arch surgery in 21 patients, 9 of whom (42.9%) underwent hemiarch replacement; in this cohort, they observed 1 operative death (11.1%) and 2 reoperations for bleeding (22.2%) (15). By comparison, we had no in-hospital deaths, reoperations for bleeding, strokes, or TIAs. Median LOS in our UHS cohort was comparable to Kim et al. (7 vs. 7 d), despite our patients being older (74 vs. 58 yr) (11). We experienced no conversions to FS, confirming that excellent exposure of the aortic arch, ascending aorta, and aortic valve can be safely achieved without visualization of the right ventricle. Moreover, UHS can be readily converted to FS if needed.

Our median UHS times were 192 min for CPB, 105 min for ACC, and 5 min for HCA, compared with Kim et al. (mean CPB 114 min; ACC 91.1 min; HCA 8.9 min) and Goebel et al. (mean CPB 168.7 min; ACC 115.6 min; HCA 26.4 min) in UHS patients (11, 15). During UHS, our ACC times were 52 min shorter (*P* = 0.03) compared with FS. This difference may reflect a stepwise progression in mastering UHS, beginning with FS, advancing to simpler UHS procedures such as UHS AVR, and ultimately progressing to complex aortic surgery, including ARR with scAAR, alongside the use of COR-KNOT® and Custodiol-HTK cardioplegia during UHS. COR-KNOT® simplifies and accelerates suture fixation, while Custodiol-HTK enables up to 2–3 h of safe myocardial ischemic arrest with a single dose (16, 17). In contrast, cold-blood potassium-based cardioplegia requires re-dosing every 15–20 min and hand-tying sutures within the confined space of a small aortic root can be cumbersome, disrupting surgical workflow and fluidity (16, 17).

To minimize bleeding, the distal anastomosis is constructed using a belt-and-suspenders approach. First, a strip of Teflon felt is incorporated into the suture line, compressing the aortic wall between the felt and the graft to reduce tension. Second, the anastomosis is externally reinforced with Teflon felt pledgets. Patients receive a 10 g loading dose of aminocaproic acid, followed by a continuous infusion at 1 g/h for the duration of the procedure (18). The right pleural space is opened to reduce the risk of cardiac tamponade. In cases of refractory bleeding after HCA, Prothrombin Complex Concentrate can be used, but we administer only one-quarter of the standard dose to limit hypercoagulable complications (18).

Kim and colleagues perform hemiarch repair via UHS under HCA without cerebral perfusion (11). In contrast, we use cerebral perfusion in all UHS hemiarch cases to reduce potential complications, address extensive pathology, and increase safe HCA time, which is particularly advantageous when utilizing a smaller incision. We use bACP for extended hemiarch replacements, particularly when the distal anastomosis is opposite the left carotid or left subclavian artery, as these procedures typically involve longer HCA times. This approach maintains continuous cerebral perfusion while keeping the operative field clear. Innominate artery cannulation is a viable alternative to right axillary cannulation, avoiding an additional incision and side-branch graft, though it may add clutter to the UHS field. For shorter anticipated HCA times, such as during open anastomosis at the level of the innominate artery, we utilize RCP during UHS.

The ideal UHS candidate is a generally healthy patient without significant comorbidities or concomitant cardiac disease, presenting for elective surgery. These patients benefit most from reduced surgical trauma, limited dissection, decreased adhesion formation, faster recovery, and improved cosmetic outcomes. In contrast, patients requiring urgent or emergent procedures (e.g., ATAAD), TAR with HCA, concomitant CABG, or with prior CABG and patent midline-crossing grafts are not UHS candidates and were excluded from this study. The need for greater operative exposure and technical complexity, especially in TAR, as well as meticulous coronary ostial inspection in ATAAD, makes FS our preferred approach in these cases.

### Limitations

This retrospective review of a large, prospectively maintained aortic registry is limited by its small sample size and potential selection bias, as it reflects a highly selected patient cohort. The exclusion of complex and urgent/emergent cases, such as TARs and ATAADs, further limits the applicability of our findings to higher-risk surgical populations. All procedures were performed by a single surgeon with specialized expertise in aortic surgery, which minimizes inter-operator variability but limits generalizability. Causes of death in both groups, as well as complete longitudinal and survival data after FS, were not reported due to indeterminate causes of death and partial follow-up among FS patients. Progress in surgical technique and perioperative management over the study period may have contributed to outcomes, especially in the more recent UHS cohort. Moreover, the non-randomized selection of surgical approach introduces the possibility of unmeasured confounding. These preliminary, hypothesis-generating findings should be validated in a larger, prospective study adequately powered to detect meaningful differences between groups.

## Conclusions

Our experience comprises a select cohort of 11 patients who underwent elective UHS hemiarch and proximal aortic replacement under HCA with bACP or RCP. There were no in-hospital deaths or postoperative occurrences of stroke, TIA, renal insufficiency, or reoperation for bleeding following UHS. Postoperative outcomes were comparable to those of the 15 patients who underwent FS, except that the ACC time was significantly shorter in the UHS group. These findings demonstrate the safety and durability of UHS in complex aortic surgery beyond the immediate postoperative period and underscore key technical aspects that may support its broader adoption. Prospective studies with larger cohorts are warranted to validate these preliminary results.

## Data Availability

The raw data supporting the conclusions of this article will be made available by the authors, without undue reservation.
